# *Tetragonia tetragonoides* (Pall.) Kuntze (New Zealand Spinach) Prevents Obesity and Hyperuricemia in High-Fat Diet-Induced Obese Mice

**DOI:** 10.3390/nu10081087

**Published:** 2018-08-14

**Authors:** Young-Sil Lee, Seung-Hyung Kim, Heung Joo Yuk, Geung-Joo Lee, Dong-Seon Kim

**Affiliations:** 1 Herbal Medicine Research Division, Korea Institute of Oriental Medicine, 1672 Yuseong-daero, Yuseong-gu, Dajeon 34054, Korea; rheeys04@kiom.re.kr (Y.-S.L.); yukhj@kiom.re.kr (H.J.Y.); 2 Institute of Traditional Medicine and Bioscience, Daejeon University, 62 Daehak-ro, Dong-gu, Daejeon 34520, Korea; sksh518@dju.kr; 3 Department of Horticulture, Chungnam National University, 99 Daehak-ro, Yuseong-gu, Daejeon 34134, Korea

**Keywords:** *Tetragonia tetragonoides* (Pall.) Kuntze, obesity, hyperuricemia, lipogenesis, fatty acid oxidation, xanthine oxidoreductase

## Abstract

*Tetragonia tetragonoides* (Pall.) Kuntze, called New Zealand spinach (NZS), is an edible plant used in salad in Western countries and has been used to treat gastrointestinal diseases in traditional medicine. We examined the anti-obesity and anti-hyperuricemic effects of NZS and the underlying mechanisms in high-fat diet (HFD)-induced obese mice. Mice were fed a normal-fat diet (NFD); high-fat diet (HFD); HFD with 75, 150, or 300 mg/kg NZS extract; or 245 mg/kg *Garcinia cambogia* (GC) extract. NZS decreased body weight gain, total white adipose tissue (WAT), liver weight, and size of adipocytes and improved hepatic and plasma lipid profiles. With NZS, the plasma levels of the leptin and uric acid were significantly decreased while the levels of the adiponectin were increased. Furthermore, NZS decreased the expression levels of adipogenesis-related genes and xanthine oxidoreductase (XOR), which is involved in uric acid production, while increasing that of proteins associated with fatty acid oxidation. UPLC analysis revealed that NZS contained 6-methoxykaempferol-3-*O*-β-d-glucosyl(1′′′→2′′)-β-d-glucopyranoside, 6-methoxykaempferol-3-*O*-β-d-glucosyl(1′′′→2′′)-β-d-glucopyranosyl-(6′′′′-caffeoyl)-7-*O*-β-d-glucopyranoside, and 6,4′-dimethoxykaempferol-3-*O*-β-d-glucosyl(1′′′→2′′)-β-d-glucopyranosyl-(6′′′′-caffeoyl)-7-*O*-β-d-glucopyranoside. These results suggest that NZS exerts anti-obesity, anti-hyperlipidemia, and anti-hyperuricemic effects in HFD-induced obese mice, which are partly explained by regulation of lipid-metabolism-related genes and proteins and decreased expression of XOR.

## 1. Introduction

Obesity is a serious public health problem that threatens human health worldwide. It results from abnormalities in energy metabolism in adipose tissue, which leads to increased fat mass in tissues, such as adipose tissue and liver tissue, and elevated lipids levels in the blood [1,2]. Obesity is associated with the development of metabolic disorders, such as type 2 diabetes, hyperlipidemia, fatty liver, hypertension, and cardiovascular disease [3,4]. Hyperuricemia is characterized by elevated blood uric acid levels, the final product of purine metabolism. Uric acid plays an important role in the development of metabolic disorders, since uric acid regulates the oxidative stress, inflammation, and proteins involved in glucose and lipid metabolism, and hyperuricemia is positively associated with increased visceral fat mass and various metabolic disorders, such as dyslipidemia, type 2 diabetes, and atherosclerosis [4,5]. Especially, uric acid production in adipose tissue is accompanied by increased xanthine oxidoreductase (XOR) activity, which degrades hypoxanthine and xanthine to uric acid. XOR expression and activity is associated with increased obesity and regulation of adipogenesis in adipose tissue [6,7]. It is considered one of the metabolic diseases linked to both obesity and various metabolic disorders, and growing attention is being paid to the relationship between obesity and uric acid metabolism [5,8].

Although pharmacological therapies are used in clinical practices to treat obesity and hyperuricemia, their use is limited by undesirable side effects [9,10]. Thus, new research on food and drugs that can overcome these limitations is needed. Therefore, increasing attention has been paid to natural products, including traditional medicines, as an alternative strategy [11].

*Tetragonia tetragonoides* (Pall.) Kuntze, called New Zealand spinach (NZS), is a perennial plant belonging to the *Aizoaceae* family. It is an edible plant used in salads in Western countries. In Korean medicine, *T. tetragonoides* has been used as a herbal medicine to treat gastrointestinal diseases, such as gastric hypersecretion, dyspepsia, gastric ulcer, and gastritis [12]. Recently, *T. tetragonoides* has been reported to improve women’s health, regulate glucose metabolism, and exert anti-inflammatory effects [13,14]. To date, there are no reports available on the effects of *T. tetragonoides* on obesity, lipid accumulation, or uric acid metabolism. In the present study, we examined the anti-obesity and anti-hyperuricemic effects of NZS and their underlying mechanisms in high-fat diet (HFD)-induced obese mice.

## 2. Materials and Methods

### 2.1. Preparation of NZS Extracts

*T. tetragonoides* (Pall.) Kuntze (NZS) leaves were collected from the ocean sand dunes of Shinan-gun, Jeonnam Province, Korea. The plants were identified by Professor Geung-Joo Lee (Department of Horticulture, Chungnam National University, Daejeon, Korea). An extract from NZS leaves (dry weight, 1 kg) was prepared with 70% ethanol under reflux in a condensation system at 85 °C for 3 h. The extract was concentrated under reduced pressure using a rotary evaporator and then lyophilized. The yield of the dried extract from the starting crude material was 11.2%.

### 2.2. Animal Study

Four-week-old male C57BL/6J mice were purchased from Daehan Biolink Co. (Eumsung, Korea). The mice were maintained under controlled temperature (23 ± 3 °C) and humidity (50%) conditions with a 12-h light/dark cycle. After 1 week of acclimatization, the mice were divided into one of the following six groups based on body weight: group 1 (normal-fat diet (NFD), *n* = 5) was fed a standard chow diet (14% fat, 21% protein, and 65% carbohydrate; Orient Bio Inc., Seongnam, Korea); group 2 (high-fat diet (HFD), *n* = 5) was fed a high-fat diet (60% fat, 20% protein, and 20% carbohydrate; rodent diet D12492, Research Diets, New Brunswick, NJ, USA); groups 3, 4, and 5 (HFD + NZS 75, 150, and 300, *n* = 5, respectively) were fed an HFD and given NZS at doses of 75, 150, and 300 mg/kg, respectively; and group 6 (HFD + *Garcinia cambogia* (GC), *n* = 5) was fed an HFD and given *Garcinia cambogia* (GC) extract at a dose of 245 mg/kg as a positive control. NZS and GC were dissolved in a vehicle (0.5% carboxylmethylcellulose) and orally administered once daily for 8 weeks. Mice in the NFD and HFD groups were administered the vehicle alone. The mice were allowed free access to water and food, and the body weights and food intake were measured every week. Food efficiency rate was calculated as (total weight gain/total food intake) × 100. The experimental design was approved by the Institutional Animal Care and Use Committee of Daejeon University, and all experiments were performed in accordance with committee guidelines.

### 2.3. Blood Collection and Biochemical Analyses of Plasma

After 8 weeks, the mice were euthanized with ether after overnight fasting. Blood samples were collected by cardiac puncture and centrifuged to obtain plasma, and separated plasma samples were stored at −80 °C until use. Plasma glucose, triglyceride (TG), non-esterified fatty acid (NEFA), total cholesterol (T-CHO), low-density lipoprotein cholesterol (LDL-CHO), and high-density lipoprotein cholesterol (HDL-CHO) were analyzed using an automatic biochemical analyzer (Hitachi-7020, Hitachi Medical, Tokyo, Japan). Plasma uric acid levels were determined with a colorimetric assay kit (BioVision, Milpitas, CA, USA). Plasma leptin and adiponectin levels were measured by immunoassay using ELISA kits (Mouse Leptin and Adiponectin/Acrp30; R&D Systems, Minneapolis, MN, USA) according to the manufacturer’s protocols and presented corrected by total white adipose tissue (WAT) weight.

### 2.4. Tissue Collection and Histological Analysis

Liver and white adipose tissue (WAT; epididymal, perirenal, mesenteric, and subcutaneous WAT) were dissected immediately, rinsed, weighed, frozen in liquid nitrogen, and stored at −80 °C until analysis. For histological examination, the liver and WAT were fixed in a 10% formalin solution and embedded in paraffin. Sections were stained with hematoxylin and eosin (H&E). Adipocyte size was determined by measuring the area taken up by 20 adipocytes in a stained section and adipocyte cell number was measured by Image J1.49 software (http://rsb.info.nih.gov/ij/download.html). To determine liver lipid accumulation, liver sections were stained with Oil-red O and hematoxylin.

### 2.5. Measurement of Liver TG Levels

Liver tissue (0.1 g) was homogenized in 95% ethanol and centrifuged. The resulting supernatant was mixed with sodium chlorate and triton X-100. TG levels were determined using a commercial kit (Triglyceride E test, Wako Pure Chemical Industries, Osaka, Japan) according to the manufacturer’s protocol.

### 2.6. Total RNA Isolation and Gene Expression Analyses

Total RNA was isolated from WAT of mice with the TRI reagent (Sigma-Aldrich Co., St. Louis, MO, USA) and reverse-transcribed to cDNA with an oligo primer using a FirstStand cDNA synthesis kit (Amersham Pharmacia, Piscataway, NJ, USA) according to the manufacturer’s protocol. The mRNA expression levels of adipogenesis-related genes were analyzed using gene-specific primers, probes, Power SYBR^®^ Green PCR Master Mix, and TaqMan^®^ Gene E-expression Master Mix (Applied Biosystems, Foster City, CA, USA) according to the manufacturer’s instructions using a real-time PCR ABI Prism 7700 system (Applied Biosystems). The primer and probe sequences are as follows: CCAAT/enhancer binding protein (C/EBP)α (GeneBank accession number: BC016892): forward, 5′-TGGACAAGAACAGCAACGAGTAC-3′ and reverse, 5′-CGGTCATTGTCACTGGTCAACT-3′; Peroxisome proliferator-activated receptor (PPAR)γ (GeneBank accession number: NM_011146): forward, 5′-CCCTGGCAAAGCATTTGTAT-3′ and reverse, 5′-GAAACTGGCACCCTTGAAAA-3′; adipocytes fatty acid-binding protein (aP2/FABP4) (GeneBank accession number: NM_024406): forward, 5′-TGGGAACCTGGAAGCTTGTCTC-3′ and reverse, 5′-GAATTCCACGCCCAGTTTGA-3′; Fatty acid synthase (FAS) (GeneBank accession number: NM_007988): forward, 5′-CTGAGATCCCAGCACTTCTTGA-3′ and reverse, 5′-GCCTCCGAAGCCAAATGAG-3′; Sterol regulatory element-binding protein (SREBP)-1c (GeneBank accession number: BC056922.1): forward, 5′-AGCCTGGCCATCTGTGAGAA-3′ and reverse, 5′-CAGACTGGTACGGGCCACAA-3′; Stearoly-CoA desaturase (SCD)-1 (GeneBank accession number: AH002082.2): forward, 5′-CATCGCCTGCTCTACCCTTT-3′ and reverse, 5′-GAACTGCGCTTGGAAACCTG-3′; Acetyl-CoA carboxylase (ACC)-1 (GeneBank accession number: NM_133360.2): forward, 5′-ATTGTGGCTCAA ACTGCAGGT-3′ and reverse, 5′-GCCAATCCACTCGAAGACCA-3′; Diglyceride acyltransferase (DGAT)-1 (GeneBank accession number: NM_10046): forward, 5′-TGCTACGACGAGTTCTTGAG-3′ and reverse, 5′-CTCTGCCACAGCATTGAGAC-3′; and GAPDH (GeneBank accession number: XM_017321385.1): forward, 5′-AAGCTGTGGCGTGATGGCC-3′ and reverse, 5′-TGGGCCCTCAGATGCCTGCT-3′. The probe for XOR (6-Carboxyfluorescein (FAM) dye) was synthesized by Applied Biosystems. All samples were normalized to the corresponding expression of *GAPDH*. The expression level of the target genes relative to their expression level in the HFD group was calculated by comparative Ct, which is defined as the cycle number at which the fluorescence signal became significantly higher than the background.

### 2.7. Western Blotting Analysis

Protein extracts were prepared using a protein extraction kit (Intron Biotechnology Inc., Seoul, Korea). Lysates were electroblotted onto a nitrocellulose membrane following separation by SDS polyacrylamide gel electrophoresis. Blotted membranes were incubated for 1 h with blocking solution (EzBlockChemi blocking solution, ATTO Corporation, Tokyo, Japan), followed by incubation overnight at 4 °C with primary antibodies against SREBP-1c, FAS, PPARα, CTP1, AMPK, p-AMPK, and GAPDH (Cell Signaling Technology, Beverley, MA, USA) and horseradish peroxidase-conjugated secondary antibodies (Santa Cruz Biotechnology, Santa Cruz, CA, USA). Membranes were washed and then developed by electro chemiluminescence (Amersham, GE Healthcare, Uppsala, Sweden). The relative signal strength of proteins and the internal control (GAPDH) was quantified for each band and the relative expression levels of quantification of proteins were calculated as a ratio to HFD expression.

### 2.8. UPLC-QT of MS Analysis

Ultra-performance liquid chromatography (UPLC) determinations were performed using an AQUITY^TM^ UPLC system (Waters Corp., Milford, MA, USA) equipped with a binary gradient system, an auto-injector, and a UV-Visible detector. Samples (2.0 μL) were separated on a BEH C_18_ column (2.1 × 100 mm, 1.7 μm) at a flow rate of 0.4 mL/min and eluted using a linear gradient of two mobile phases containing 0.1% formic acid (A: water; B: acetonitrile). A chromatographic gradient was optimized as follows: 0 min, 10% B; 0–8 min, 10–25% B; 8–11 min, 25–90% B; 11–12 min, 90–100% B; 12–13.3 min, 100% B; and 13.4 min, back to 100–10% B. Mass spectrometry was performed using a quadrupole time-of-flight mass spectrometer (Xevo G2 QT of, Waters Corp.) equipped with an electrospray ionization (ESI) interface in the negative ion mode. It was operated using the following parameters: cone voltage 40 V, capillary voltage 2500 V, source temperature 110 °C, and desolvation temperature 350 °C. A sprayer with a reference solution of leucine-enkephalin ([M − H]^−^ *m*/*z* 554.2615) was used as the lock mass. All the extraction and chromatographic solvents were LC/GC-MS grade for analysis (J. T. Baker, Phillipsburg, NJ, USA).

### 2.9. Statistical Analysis

Data are expressed as the mean ± standard error of the mean (SEM). Differences between treatment groups were analyzed by one-way ANOVA and Dunnett’s multiple comparison tests using Prism 7.0 software (GraphPad Software Inc., San Diego, CA, USA). A *p*-value ≤ 0.05 was considered statistically significant.

## 3. Results

### 3.1. Effects of NZS on Body Weight Gain, Food Intake Rate, and Food Efficiency Ratio

**Figure 1:**
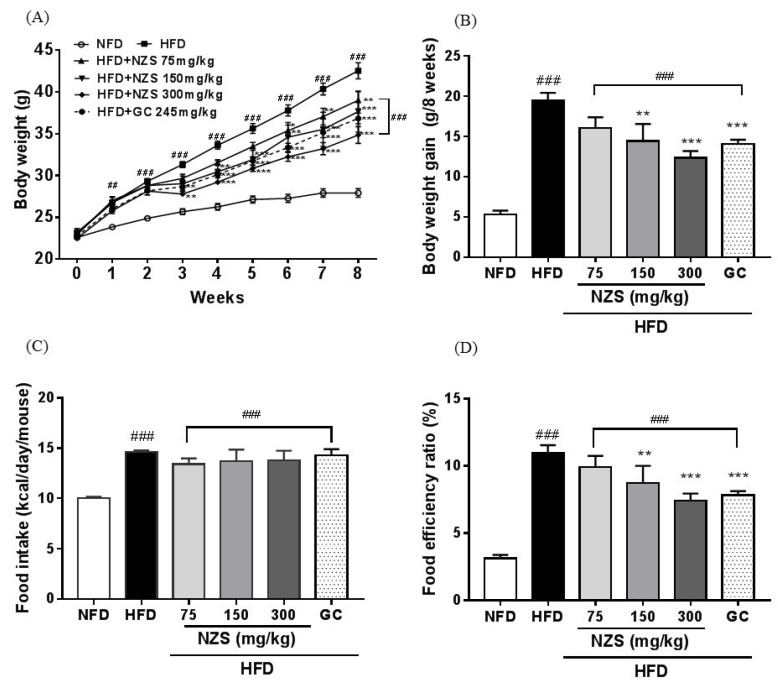
Effects of *Tetragonia tetragonoides* (Pall.) Kuntze extract (New Zealand spinach, NZS) on body weight gain and food intake in high-fat diet (HFD)-induced obese mice. (**A**) Body weight, (**B**) body weight gain, (**C**) food intake rate, and (**D**) food efficiency ratio. NFD: normal-fat diet; GC: *Garcinia cambogia* extract. Values are expressed as mean ± standard error of the mean (SEM) (*n* = 5). ^##^ *p* < 0.01 and ^###^ *p* < 0.005 versus the NFD group; ** *p* < 0.01 and *** *p* < 0.005 versus the HFD group.

Body weight and body weight gain were higher in the HFD group than in the NFD group. The body weight gains of the HFD + NZS 150 and 300 groups and the GC group were significantly lower than that of the HFD group (Figure 1A,B). The food intake calories were not significantly different between the treated groups (Figure 1C), but the NFD, HFD + NZS 150 and 300, and GC groups had decreased food efficiency ratios as compared to the HFD group (Figure 1D).

### 3.2. Effects of NZS on WAT Weight and Adipocyte Size

**Figure 2:**
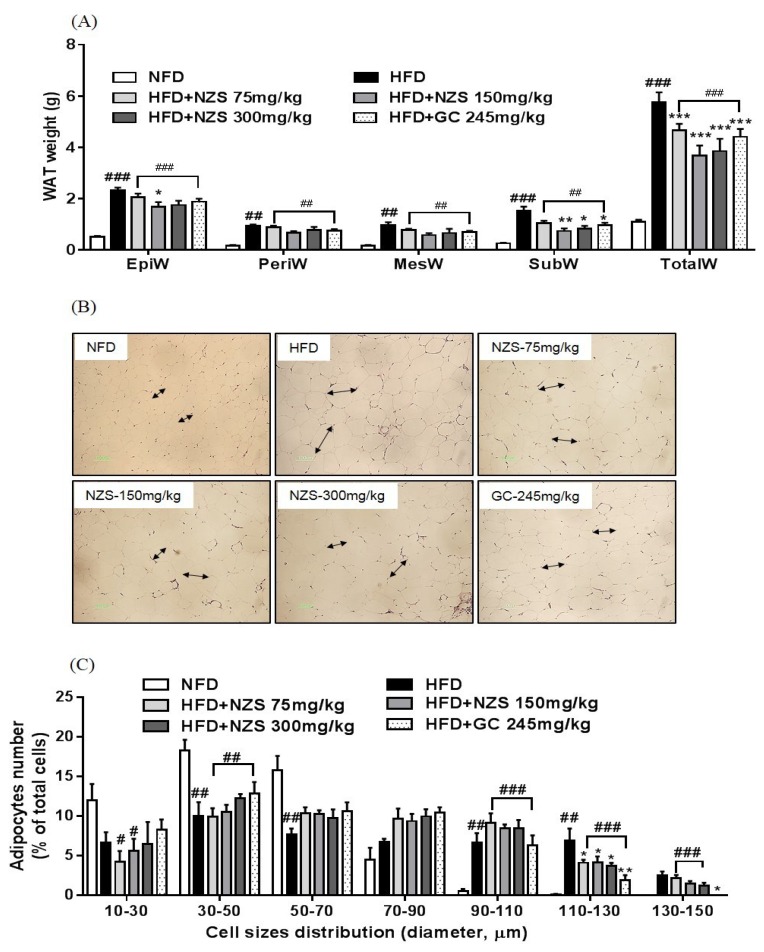
Effects of NZS on white adipose tissue (WAT) weight and histological analysis in HFD-induced obese mice. (**A**) WAT, (**B**) H&E staining, and (**C**) distribution of cell diameters. EpiW: epididymal WAT; PeriW: perirenal WAT; MesW: mesenteric WAT; SubW: inguinal WAT; TotalW: EpiW + PeriW + MesW + SubW. NFD: normal-fat diet; HFD: high-fat diet; NZS: *Tetragonia tetragonoides* (Pall.) Kuntze extract (New Zealand spinach); GC: *Garcinia cambogia* extract. Values are expressed as mean ± SEM (*n* = 5). ^#^ *p* < 0.05, ^##^ *p* < 0.01, and ^###^ *p* < 0.005 versus the NFD group; * *p* < 0.05, ** *p* < 0.01, and *** *p* < 0.005 versus the HFD group.

As shown in Figure 2A, the epididymal, perirenal, mesenteric, abdominal subcutaneous, and total WAT weights in the HFD groups were higher than those in the NFD group. The epididymal WAT weight was significantly lower in the HFD + NZS 150 group. Abdominal subcutaneous WAT weights were significantly reduced in the HFD + NZS 150 and 300 groups. The total WAT weight was significantly reduced in all three HFD + NZS groups and the GC group as compared to the HFD group. The sizes of adipocytes were considerably smaller in the NFD group, all three HFD + NZS groups, and the GC group as compared to the HFD group, and shifted in the distribution towards reduced cell diameters in the HFD + NZS groups and GC group (Figure 2B,C).

### 3.3. Effects of NZS on Adipogenesis-Related Gene Expression in WAT

**Figure 3:**
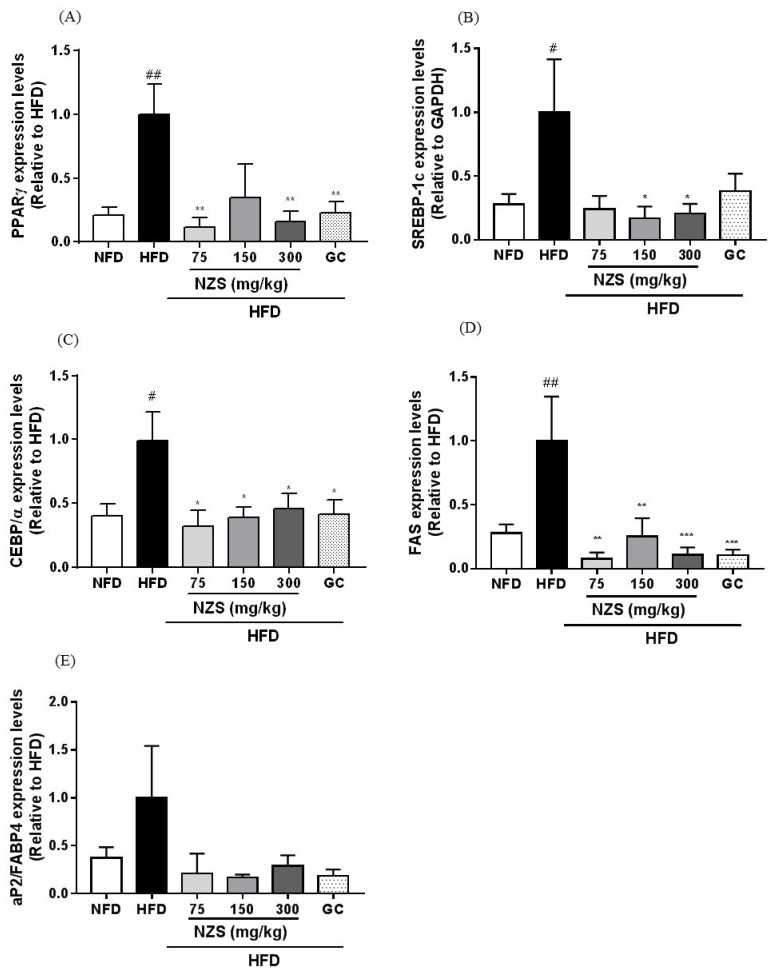
Effects of NZS on the expression levels of adipogenesis-related genes in WAT. mRNA expression levels of (**A**) PPARγ, (**B**) SREBP-1c, (**C**) CEBP/α, (**D**) FAS, and (**E**) aP2/FABP4. NFD: normal-fat diet; HFD: high-fat diet; NZS: *Tetragonia tetragonoides* (Pall.) Kuntze extract (New Zealand spinach); GC: *Garcinia cambogia* extract. Values are expressed as mean ± SEM (*n* = 5). ^#^ *p* < 0.05 and ^##^ *p* < 0.01 versus the NFD group; * *p* < 0.05, ** *p* < 0.01, and *** *p* < 0.005 versus the HFD group.

CEBP/α, PPARγ, SREBP-1c, and FAS mRNA expression levels were elevated in the HFD group compared to the NFD group. PPARγ mRNA expression levels in the HFD + NZS 75 and 300 groups and the GC group were lower than those in the HFD group (Figure 3A). SREBP-1c mRNA expression levels were significantly reduced in the HFD + NZS 150 and 300 groups (Figure 3B). CEBP/α and FAS mRNA expression levels decreased in all three HFD + NZS groups and the GC group (Figure 3C,D). aP2/FABP4 mRNA expression levels did not significantly differ between treated groups (Figure 3E).

### 3.4. Effects of NZS on Plasma Adipokine Levels

**Figure 4:**
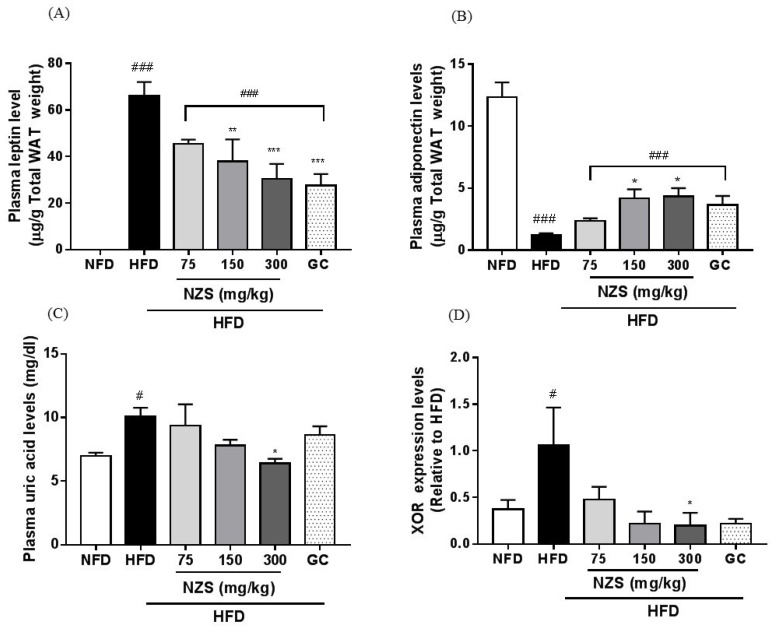
Effects of NZS on adipokine and plasma uric acid levels in HFD-induced obese mice. (**A**) Leptin, (**B**) adiponectin, (**C**) plasma uric acid levels, and (**D**) xanthine oxidoreductase (XOR) gene expression. NFD: normal-fat diet; HFD: high-fat diet; NZS: *Tetragonia tetragonoides* (Pall.) Kuntze extract (New Zealand spinach); GC: *Garcinia cambogia* extract. Values are expressed as mean ± SEM (*n* = 5). ^#^ *p* < 0.05 and ^###^ *p* < 0.005 versus the NFD group; * *p* < 0.05, ** *p* < 0.01, and *** *p* < 0.005 versus the HFD group.

Plasma leptin levels were significantly lower in the NFD group, the HFD + NZS 150 and 300 groups, and the GC group, whereas plasma adiponectin levels were significantly higher in the NFD and HFD + NZS 150 and 300 groups compared to the HFD group (Figure 4A,B).

### 3.5. Effects of NZS on Plasma Uric Acid Levels and XOR Gene Expression

Plasma uric acid levels were higher in the HFD group than in the NFD group. They tended to decrease in the HFD + NZS groups and the GC group but decreased significantly only in the HFD + NZS 300 group (Figure 4C). As shown in Figure 4D, XOR mRNA expression levels increased in the WAT of the HFD group compared to the NFD group. XOR mRNA expression trended towards a decrease in the HFD + NZS and GC groups and were significantly decreased in the HFD + NZS 300 group as compared to the HFD group.

### 3.6. Effects of NZS on Biochemical Plasma Parameters

**Figure 5:**
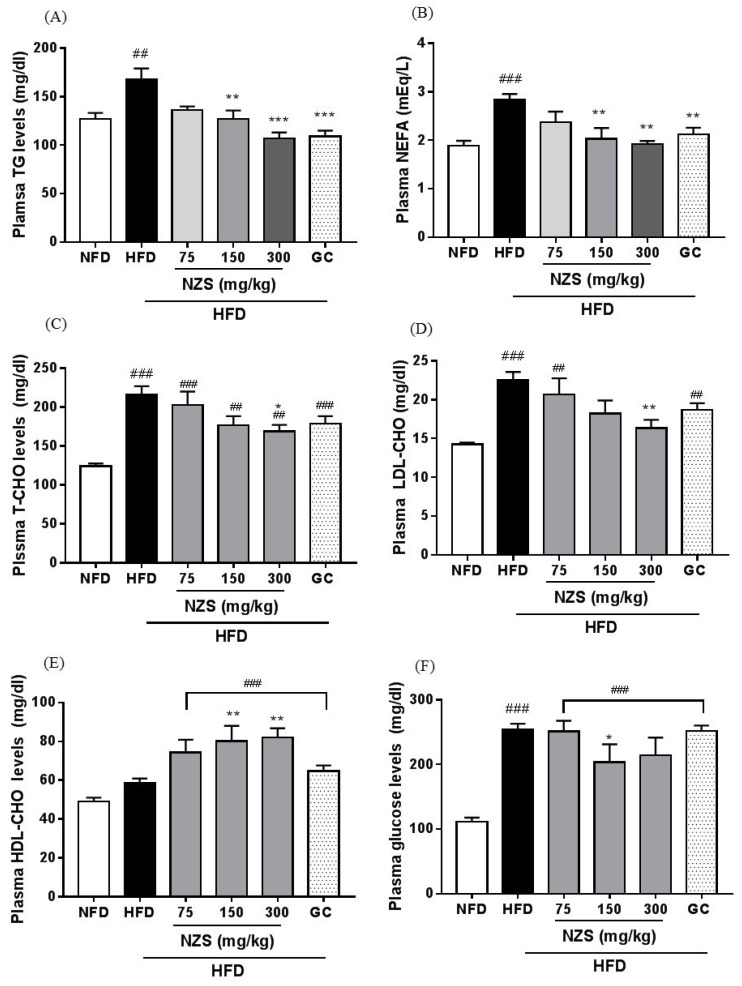
Effects of NZS on biochemical parameters in HFD-induced obese mice. (**A**) TG, (**B**) NEFA, (**C**) T-CHO, (**D**) LDL-CHO, (**E**) HDL-CHO, and (**F**) glucose. NFD: normal-fat diet; HFD: high-fat diet; NZS: *Tetragonia tetragonoides* (Pall.) Kuntze extract (New Zealand spinach); GC: *Garcinia cambogia* extract. Values are expressed as mean ± SEM (*n* = 5). ^##^ *p* < 0.01 and ^###^ *p* < 0.005 versus the NFD group; * *p* < 0.05, ** *p* < 0.01, and *** *p* < 0.005 versus the HFD group.

Plasma TG, NEFA, T-CHO, LDL-CHO, and glucose levels in the HFD group were higher than those in the NFD group. Plasma TG and NEFA levels were significantly reduced in the HFD + NZS 150 and 300 groups and the GC group (Figure 5A,B). T-CHO and LDL-CHO levels were significantly lower in the HFD + NZS 300 group, whereas HDL-CHO levels were higher in the HFD + NZS 150 and 300 groups compared with the HFD group (Figure 5C–E). Plasma glucose levels significantly decreased in the HFD + NZS 150 group compared to the HFD group (Figure 5F).

### 3.7. Effects of NZS on Liver and Kidney Weight and Hepatic Lipid Accumulation

**Figure 6:**
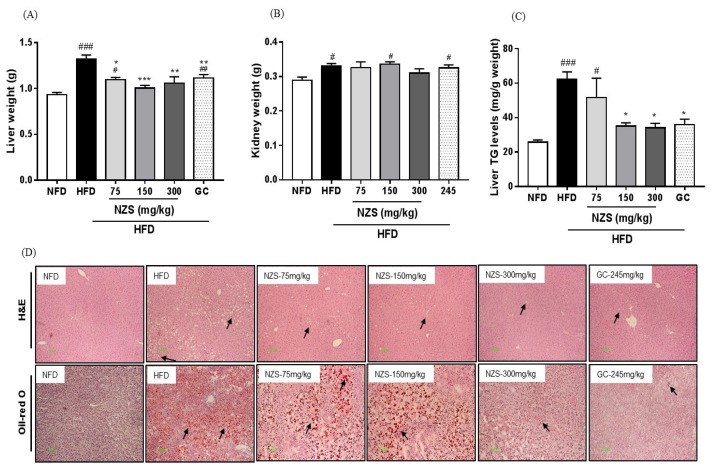
Effects of NZS on liver weight and hepatic lipid accumulation in HFD-induced obese mice. (**A**) Liver weight, (**B**) kidney weight, (**C**) liver TG levels, and (**D**) liver H&E and Oil-red O staining. NFD: normal-fat diet; HFD: high-fat diet; NZS: *Tetragonia tetragonoides* (Pall.) Kuntze extract (New Zealand spinach); GC: *Garcinia cambogia* extract. Values are expressed as mean ± SEM (*n* = 5). ^#^ *p* < 0.05, ^##^ *p* < 0.01, and ^###^ *p* < 0.005 versus the NFD group; * *p* < 0.05, ** *p* < 0.01, and *** *p* < 0.005 versus the HFD group.

The liver weight was significantly lower in the NFD group, all three HFD + NZS groups, and the GC group compared with the HFD group (Figure 6A). The kidney weight was significantly lower in the NFD group, but did not differ in the HFD + NZS groups and the GC group compared with the HFD group (Figure 6B). Liver TG levels were significantly lower in the HFD + NZS 150 and 300 groups and the GC group than those in the HFD group (Figure 6C). Consistent with this result, lipid accumulation as assessed by histological examination and Oil-red O staining in the livers of the NFD, HFD + NZS, and GC groups were lower than that in the HFD group (Figure 6D).

### 3.8. Effects of NZS on Lipid-Metabolism-Related Genes and Protein Expression in the Liver

**Figure 7:**
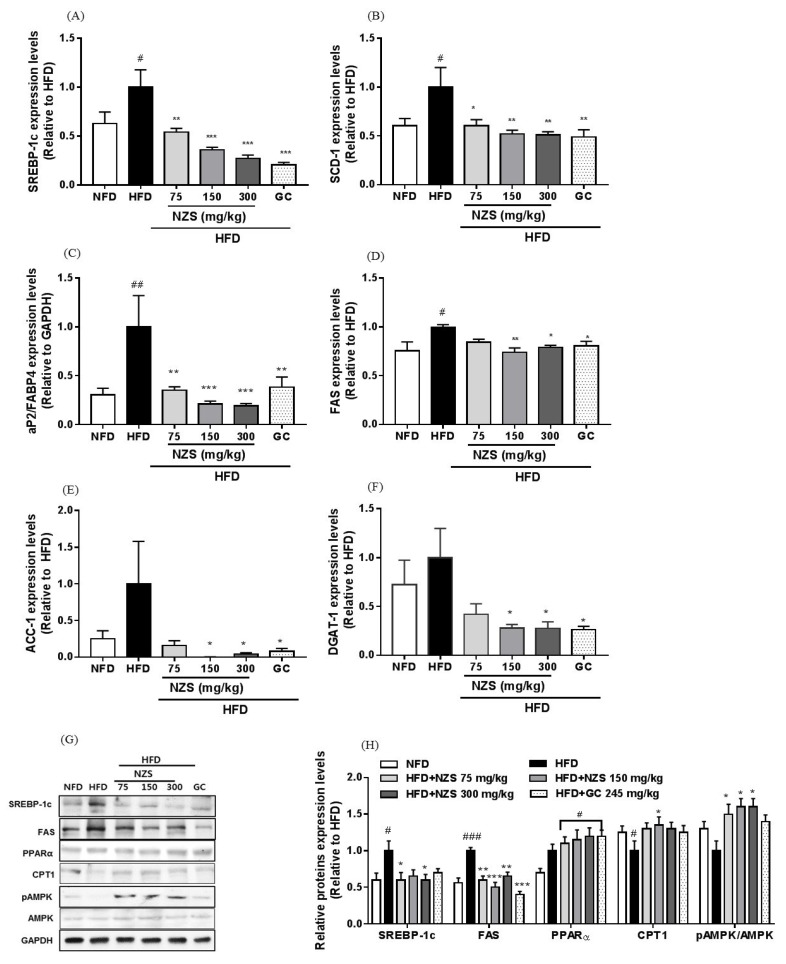
Effects of NZS on the expression levels of lipid-metabolism-related genes and proteins in the liver. mRNA expression levels of (**A**) SREBP-1c, (**B**) SCD-1, (**C**) aP2/FABP4, (**D**) FAS, (**E**) ACC-1, (**F**) DGAT-1, (**G**) representative protein expression, and (**H**) densitometric analyses of protein expression levels. NFD: normal-fat diet; HFD: high-fat diet; NZS: *Tetragonia tetragonoides* (Pall.) Kuntze extract (New Zealand spinach); GC: *Garcinia cambogia* extract. Values are expressed as mean ± SEM (*n* = 5). ^#^ *p* < 0.05, ^##^ *p* < 0.01, and ^###^ *p* < 0.005 versus the NFD group; * *p* < 0.05, ** *p* < 0.01, and *** *p* < 0.005 versus the HFD group.

SREBP-1c, SCD-1, aP2/FABP4, and FAS mRNA expression levels increased in the HFD group compared to the NFD group. They were significantly decreased in all three HFD + NZS groups and the GC group compared with those in the HFD group (Figure 7A–C). FAS, ACC-1, and DGAT-1 mRNA expression levels decreased in the HFD + NZS 150 and 300 groups and the GC group compared to those in the HFD group (Figure 7D–E). Similar to gene expression levels, SREBP-1c and FAS protein levels were reduced in all three HFD + NZS groups and the GC group. In addition, CPT1 protein expression levels increased in the HFD + NZS 150 group and AMPK phosphorylation levels increased in all three HFD + NZS groups (Figure 7G,H).

### 3.9. Identification of Major Compounds in NZS by UPLC-QT of MS

**Figure 8:**
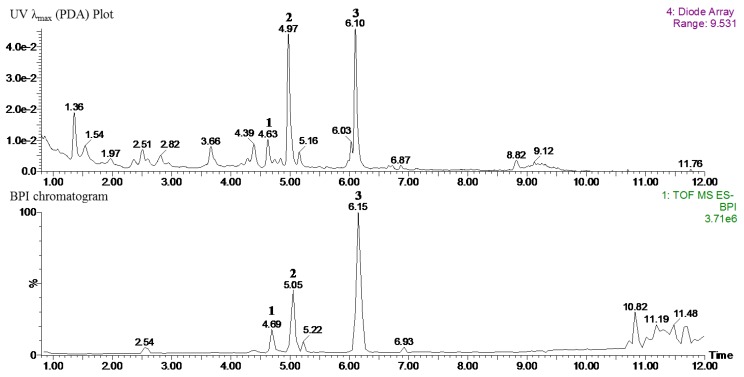
UPLC-PDA-QT of MS chromatograms of NZS extract: 6-methoxykaempferol-3-*O*-β-d-glucosyl(1′′′→2′′)-β-d-glucopyranoside (**1**), 6-methoxykaempferol-3-*O*-β-d-glucosyl(1′′′→2′′)-β-d-glucopyranosyl-(6′′′′-caffeoyl)-7-*O*-β-d-glucopyranoside (**2**), and 6,4′-dimethoxykaempferol-3-*O*-β-d-glucosyl(1′′′→2′′)-β-d-glucopyranosyl-(6′′′′-caffeoyl)-7-*O*-β-d-glucopyranoside (**3**).

As shown in Figure 8, the three major phytochemicals were identified by HR-MS analysis. The details for the identification were as follows. Compounds (**1**–**3**) were determined to be 6-methoxykaempferol derivatives belonging to the glycosylated flavonol class of representative flavonoids in NZS. Peak **1** had an [M − H]^-^ at *m*/*z* 639.1551 (−1.6 ppm error) and fragment ions at *m*/*z* 315 for the aglycon 6-methoxykaempferol (loss of 324 amu for glucosyl-glucoside) and 299 (loss of 340 amu for glucosyl-glucoside-H-H_2_O). Peak **2** had an [M − H]^−^ at *m*/*z* 963.2426 (2.1 ppm error) and fragment ions at *m*/*z* 639 (loss of 324 amu for caffeoyl-glucoside-H-H_2_O) and 315 for the aglycon 6-methoxykaempferol (loss of 648 amu for caffeoyl-glucoside + glucosyl-glucoside). Peak **3** had an [M − H]^−^ at *m*/*z* 977.2565 (0.2 ppm error) and fragment ions at *m*/*z* 639 (loss of 324 amu for caffeoyl-glucoside-H-H_2_O) and 315 for the aglycon 6-methoxykaempferol (loss of 648 amu for caffeoyl-glucoside + glucosyl-glucoside). On the basis of this information, peaks **1**–**3** were assigned to 6-methoxykaempferol-3-*O*-β-d-glucosyl (1′′′→2′′)-β-d-glucopyranoside (**1**), 6-methoxykaempferol-3-*O*-β-d-glucosyl (1′′′→2′′)-β-d-glucopyranosyl-(6′′′′-caffeoyl)-7-*O*-β-d-glucopyranoside (**2**), and 6,4′-dimethoxykaempferol-3-*O*-β-d-glucosyl (1′′′→2′′)-β-d-glucopyranosyl-(6′′′′-caffeoyl)-7-*O*-β-d-glucopyranoside (**3**), well-known compounds in NZS. The tentative identification of the three main compounds (**1**–**3**) were characterized using spectroscopic data, including HR-MS (accurate mass in negative mode) or HR MS/MS spectra (fragmentation pattern) and UV/Vis spectra (absorption maxima), in comparison with published literature [12] (Supplementary Materials, Figures S1–S3).

## 4. Discussion

In the present study, we examined the effects of NZS on obesity, hyperlipidemia, and hyperuricemia and analyzed the underlying mechanisms in HFD-induced obese mice. We found that NZS reduced body weight gain, WAT weight, and adipocyte size in histological analyses. In addition, NZS reduced the food efficiency ratio without changing the food intake rate. These results suggest that reductions in body weight gain caused by NZS are associated with reduced WAT weight independent of food intake. Furthermore, we observed that NZS reduced expression levels of adipogenesis-related genes, such as PPARγ, C/EBPα, SREBP-1c, FAS, and aP2/FABP4, in the WAT of HFD-induced obese mice. Adipocyte differentiation and lipid accumulation have important roles in the development of obesity [15]. Since adipocyte differentiation is coordinated by a complex network of transcription factors, such as C/EBPα and PPARγ, their maintenance is important in the progression of adipogenesis. In addition, the expression of adipogenesis genes induced by transcription factors, including aP2/FABP4, and FAS, leads to increased TG accumulation in the terminal phase of differentiation [16]. Thus, a reduction of adiposity is associated with inhibition of adipogenesis. Therefore, our results suggest that NZS inhibited fat accumulation in the WAT through reduced expression of adipogenesis-related genes, indicating that NZS has anti-obesity effects. On the other hand, the number of adipocytes as well as the size reduction of adipocytes can determine the adipose tissue mass. It is determined by adipocyte differentiation and apoptosis [17]. Consequently, the effect of NZS on adipocyte cell death needs to be investigated.

Leptin and adiponectin regulate the lipid and glucose metabolism and their plasma levels are positively and negatively related to increased fat mass, respectively [18,19]. In the present study, NZS decreased plasma leptin levels and increased adiponectin levels, which is supported by the decreased WAT weights and adipocyte size in the histological analyses. These observations suggest that the regulation of plasma leptin and adiponectin levels by NZS may be attributed to decreased fat accumulation and adipocyte size in WAT.

Obesity is accompanied by fatty liver and hyperlipidemia. Fatty acids secreted from adipose tissue enter the liver to synthesize fatty acids and TG, which may cause fatty liver and hyperlipidemia by changing the lipid profile in the plasma [20]. In our study, NZS decreased liver weight and inhibited liver TG accumulation as determined by histological analysis, including H&E and Oil-red O staining. In addition, NZS reduced plasma TG, NEFA, T-CHO, and LDL-CHO levels, while it increased plasma HDL-CHO levels. These results suggest that NZS can efficiently regulate TG accumulation in liver and plasma lipid profiles, indicating that NZS may ameliorate fatty liver and hyperlipidemia. Especially, HDL-CHO is considered to benefit cholesterol and increased HDL-CHO levels can attenuate atherosclerosis since less cholesterol is available to attach to blood vessels. Therefore, NZS may have a beneficial effect on atherosclerosis. Additionally, as mentioned above, heightened fatty acid levels in the blood caused by HFD can lead to changes in hepatic lipid metabolism by de novo lipogenesis in the liver [19]. SREBP-1c, a transcription factor, plays a critical role in lipid metabolism via the regulation of fatty acid synthesis and uptake and modulation of TG-synthesizing genes. In the present study, NZS reduced expression levels of the lipogenesis-related genes and proteins SREBP-1c, FAS, aP2/FABP4, SCD-1, ACC-1, and DGAT-1. NZS also increased CPT1 protein expression and AMPK phosphorylation levels in the liver, which are involved in fatty acid oxidation [21,22]. These results indicate that inhibition of fat accumulation in the liver by NZS may be accompanied by reduced lipogenesis and increased fatty acid oxidation. In addition, NZS lowered plasma glucose levels. As mentioned above, NZS increased plasma adiponectin and AMPK activation. Adiponectin regulates glucose and lipid metabolism by AMPK activation, which regulates glucose and lipid metabolism by stimulating fatty acid oxidation and glucose uptake, but also inhibiting lipogenesis and glucose production in insulin target tissues [23,24]. This result indicates that NZS may have a beneficial effect on insulin resistance; however, the effects of NZS on insulin resistance need to be further investigated with methods such as insulin level analysis, homeostatic model assessment for insulin resistance, and a glucose tolerance test.

Interestingly, it has been reported recently that obesity and metabolic disorders are associated with hyperuricemia. In general, uric acid is mainly produced in the liver and primarily excreted into the urine [25,26]. According to recent reports, adipose tissue can also produce and secrete uric acid [10,27], and increased visceral fat mass is positively correlated with plasma uric acid levels [5,28]. The production of uric acid in adipose tissue is regulated by XOR, an enzyme that catalyzes purine degradation, such as hyperxanthine and xanthine, to uric acid. Additionally, the xanthine oxidase inhibitor febuxostat and knockdown of XOR by siRNA inhibit uric acid production in adipocytes [6,7]. XOR expression and activity were higher in adipose tissue of obese mice and XOR overexpression increased PPARγ activation and adipogenesis in adipocytes, whereas XOR^-/-^ mice inhibited adiposity [6,7]. Those reports demonstrate that XOR expression and activity in adipose tissue may play a role in the hyperuricemia linked to obesity. Similarly, in our study, NZS lowered plasma uric acid levels and reduced XOR gene expression, as well as PPARγ gene expression levels, in adipose tissues. These observations indicate that NZS contributes to a decrease in plasma uric acid levels in an obese state, which may be explained by the reduction in production and secretion of uric acid through decreased XOR expression in adipose tissues. Based on these results and other reports, NZS may improve adiposity and hyperuricemia in part by regulating the relationship between XOR and adipogenesis. However, further studies are needed to examine the effects of NZS on the relationships between XOR, adipogenesis, and uric acid production in adipocytes, and adipose tissue. On the other hand, it has been reported that there are species differences in purine metabolism between human and experimental animals. Unlike humans, experimental animals have an uricase enzyme that degrades uric acid to allantoin, leading to reduce the serum uric acid levels, which is a limitation of this research [29]. However, this suggests that humans are susceptible to hyperuricemia. Therefore, anti-hyperuricemic effects by NZS may be beneficial to improve hypericemia with obesity and metabolic disorders.

In our study, a high dose of NZS reduced the plasma uric acid and cholesterol in HFD-induced obese mice. Extrapolation of animal doses into human doses can be calculated based on body surface area [30], and the 300 mg/kg dose used in this study would equal about 1500 mg/day in a 60-kg human, which implies that the effect of NZS may be expected when taken at a dose of 1500 mg/kg in humans.

According to the tentative identification of phytochemicals, 6-methoxykaempferol-3-*O*-β-d-glucosyl(1′′′→2′′)-β-d-glucopyranoside (**1**), 6-methoxykaempferol-3-*O*-β-d-glucosyl (1′′′→2′′)-β-d-glucopyranosyl-(6′′′′-caffeoyl)-7-*O*-β-d-glucopyranoside (**2**), and 6,4′-dimethoxykaempferol-3-*O*-β-d-glucosyl (1′′′→2′′)-β-d-glucopyranosyl-(6′′′′-caffeoyl)-7-*O*-β-d-glucopyranoside (**3**) are the main components of the 70% ethanol extraction of NZS. As previously reported, kaempferol, caffeic acid, and glycosylated kaempferol have been shown to prevent obesity and ameliorate hyperlipidemia in vitro and in vivo [31,32,33]. However, glycosylated caffeic-acid-linked kaempferol glucosides, such as compounds **2** and **3**, have not been reported to have beneficial effects against obesity and related metabolic disorders. Therefore, we suggest the possibility that the most abundant compound, **3** (6,4′-dimethoxykaempferol-3-*O*-β-d-glucosyl (1′′′→2′′)-β-d-glucopyranosyl-(6′′′′-caffeoyl)-7-*O*-β-d-glucopyranoside), in NZS not only contributes to the anti-obesity and anti-hyperuricemia effects as a bioactive compound, but also that these compounds may exert synergistic or additive effects. However, further investigation into the effects of these compounds on obesity and related metabolic disorders is required.

## 5. Conclusions

We found that NZS reduced body weight gain, WAT and adipocyte size, liver weight, hepatic TG accumulation, and plasma uric acid levels and changed plasma lipid profiles, suggesting that NZS has anti-obesity, anti-hyperlipidemic, and anti-hyperuricemic effects. These effects of NZS were associated with a reduced expression of XOR and adipogenesis-related genes and increased the number of proteins involved in fatty acid oxidation. To our knowledge, this is the first report on the anti-obesity and anti-hyperuricemia activities and mechanisms of NZS. Our results suggest that NZS could be used for the prevention and treatment of obesity, hyperlipidemia, and hyperuricemia.

## Supplementary Materials

The following are available online at http://www.mdpi.com/2072-6643/10/8/1087/s1, Figure S1: Spectroscopic data (UV-VIS, MS, MS^2^, and HRESI-MS) of compound **1**; Figure S2: Spectroscopic data (UV-VIS, MS, MS^2^, and HRESI-MS) of compound **2**; Figure S3: Spectroscopic data (UV-VIS, MS, MS^2^, and HRESI-MS) of compound **3**.
