# Supplementary material for: Tetragonia tetragonoides (Pall.) Kuntze (New Zealand Spinach) Prevents Obesity and Hyperuricemia in High-Fat Diet-Induced Obese Mice
Source: Nutrients. 2018 Aug 14;10(8):1087. doi: 10.3390/nu10081087 (PMC6116159; doi:10.3390/nu10081087)
Supplement: Supplementary file 1 [file nutrients-10-01087-s001.pdf]

SUPPLEMENTARY MATERIAL

Tetragonia tetragonoides (Pall.) Kuntze (New Zealand spinach) prevents obesity and hyperuricemia in high-fat diet-induced obese mice

Figure S1. Spectroscopic data (UV-VIS, MS, MS², and HRESI-MS) of compound 1.

<u>**Tentative Identification of compound 1**</u>

※ Peak 01 : 6-methoxykaemferol-3-*O*-β-D-glucosyl(1'''→2'')-β-D-glucopyranoside (**1**)

[UV spectrum, MS1 Chromatogram, MS2 Chromatogram]

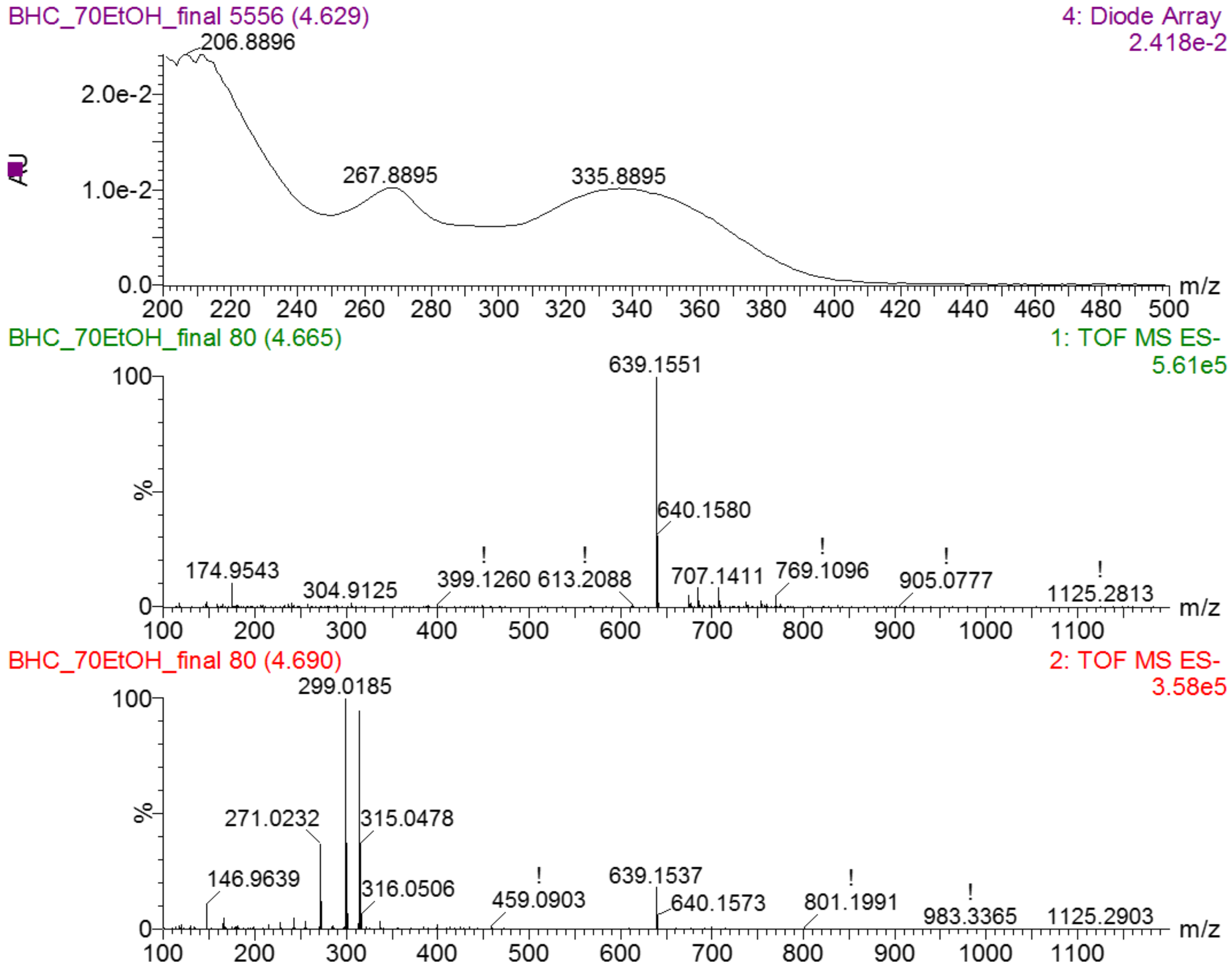

[Elemental Composition]

Single Mass Analysis
Tolerance = 10.0 PPM / DBE: min = -1.5, max = 50.0
Element prediction: Off
Number of isotope peaks used for i-FIT = 3
Monoisotopic Mass, Even Electron Ions
200 formula(e) evaluated with 3 results within limits (up to 50 best isotopic matches for each mass)
Elements Used:

Mass	Calc. Mass	mDa	PPM	DBE	Formula	i-FIT	i-FIT Norm	Fit Conf %	C	H	O
639.1551	639.1561	-1.0	-1.6	13.5	C28 H31 O17	722.4	0.000	100.00	28	31	17
	639.1503	4.8	7.5	22.5	C35 H27 O12	735.9	13.459	0.00	35	27	12
	639.1596	-4.5	-7.0	35.5	C46 H23 O4	740.3	17.874	0.00	46	23	4

Figure S2. Spectroscopic data (UV-VIS, MS, MS^2, and HRESI-MS) of compound 2.

<u>**Tentative Identification of compound 2**</u>

※ Peak 02 : 6-methoxykaemferol-3-*O*-β-D-glucosyl(1'''→2'')-β-D-glucopyranosyl-(6''''-caffeoyl)-7-*O*-β-D-glucopyranoside (**2**)

[UV spectrum, MS1 Chromatogram, MS2 Chromatogram]

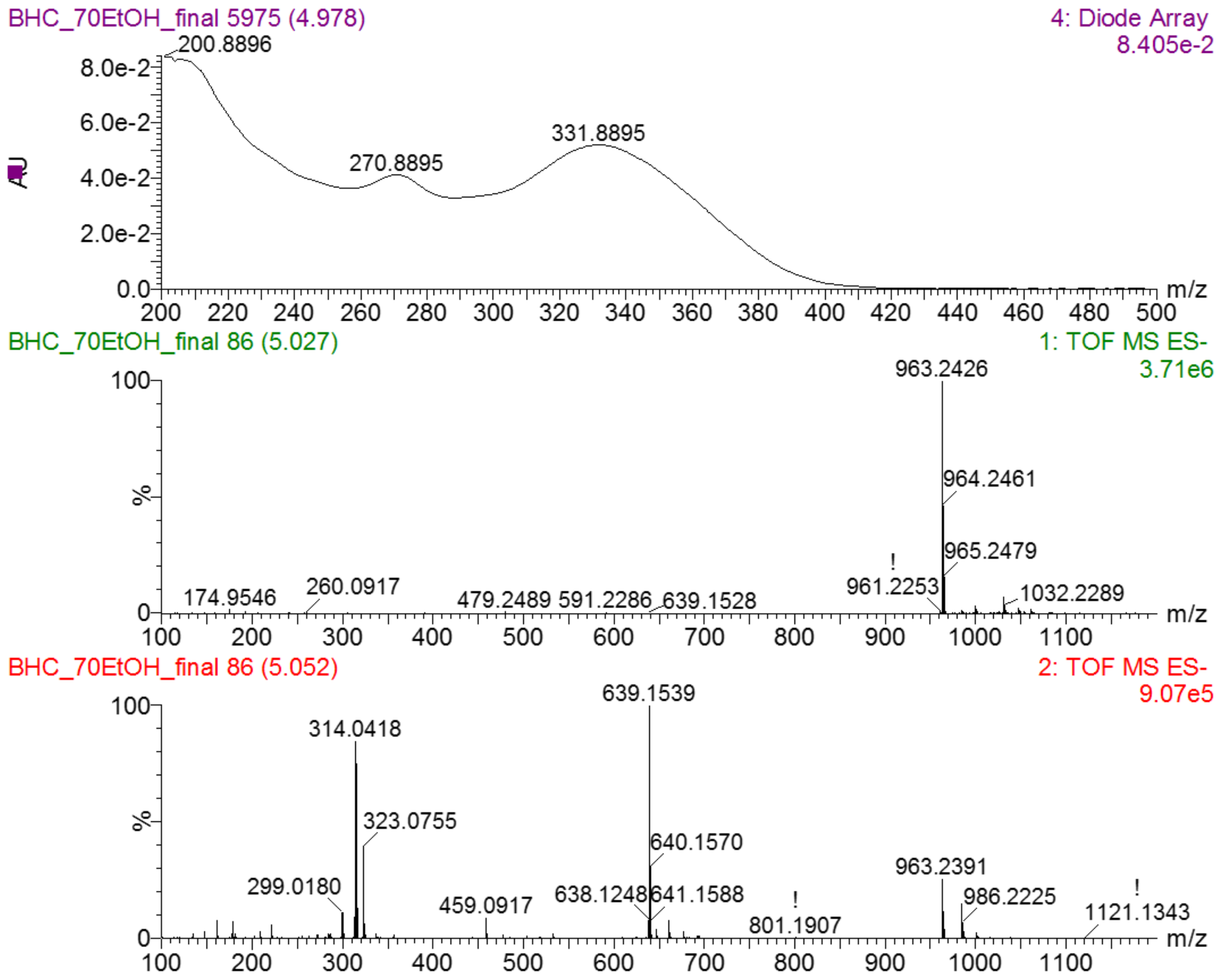

[Elemental Composition]

Single Mass Analysis
Tolerance = 10.0 PPM / DBE: min = -1.5, max = 50.0
Element prediction: Off
Number of isotope peaks used for i-FIT = 3
Monoisotopic Mass, Even Electron Ions
419 formula(e) evaluated with 6 results within limits (up to 50 best isotopic matches for each mass)
Elements Used:

Mass	Calc. Mass	mDa	PPM	DBE	Formula	i-FIT	i-FIT Norm	Fit Conf %	C	H	O
963.2426	963.2406	2.0	2.1	20.5	C43 H47 O25	678.6	0.002	99.77	43	47	25
	963.2465	-3.9	-4.0	11.5	C36 H51 O30	684.8	6.160	0.21	36	51	30
	963.2348	7.8	8.1	29.5	C50 H43 O20	688.0	9.357	0.01	50	43	20
	963.2442	-1.6	-1.7	42.5	C61 H39 O12	689.3	10.683	0.00	61	39	12
	963.2500	-7.4	-7.7	33.5	C54 H43 O17	689.6	10.961	0.00	54	43	17
	963.2371	5.5	5.7	-1.5	C25 H55 O38	689.9	11.270	0.00	25	55	38

Figure S3. Spectroscopic data (UV-VIS, MS, MS[2], and HRESI-MS) of compound 3

Tentative Identification of compound **3**

※ Peak 03 : 6,4'-dimethoxykaemferol-3-*O*-β-D-glucosyl(1'''→2'')-β-D-glucopyranosyl-(6''''-caffeoyl)-7-*O*-β-D-glucopyranoside (**3**)

[UV spectrum, MS1 Chromatogram, MS2 Chromatogram]

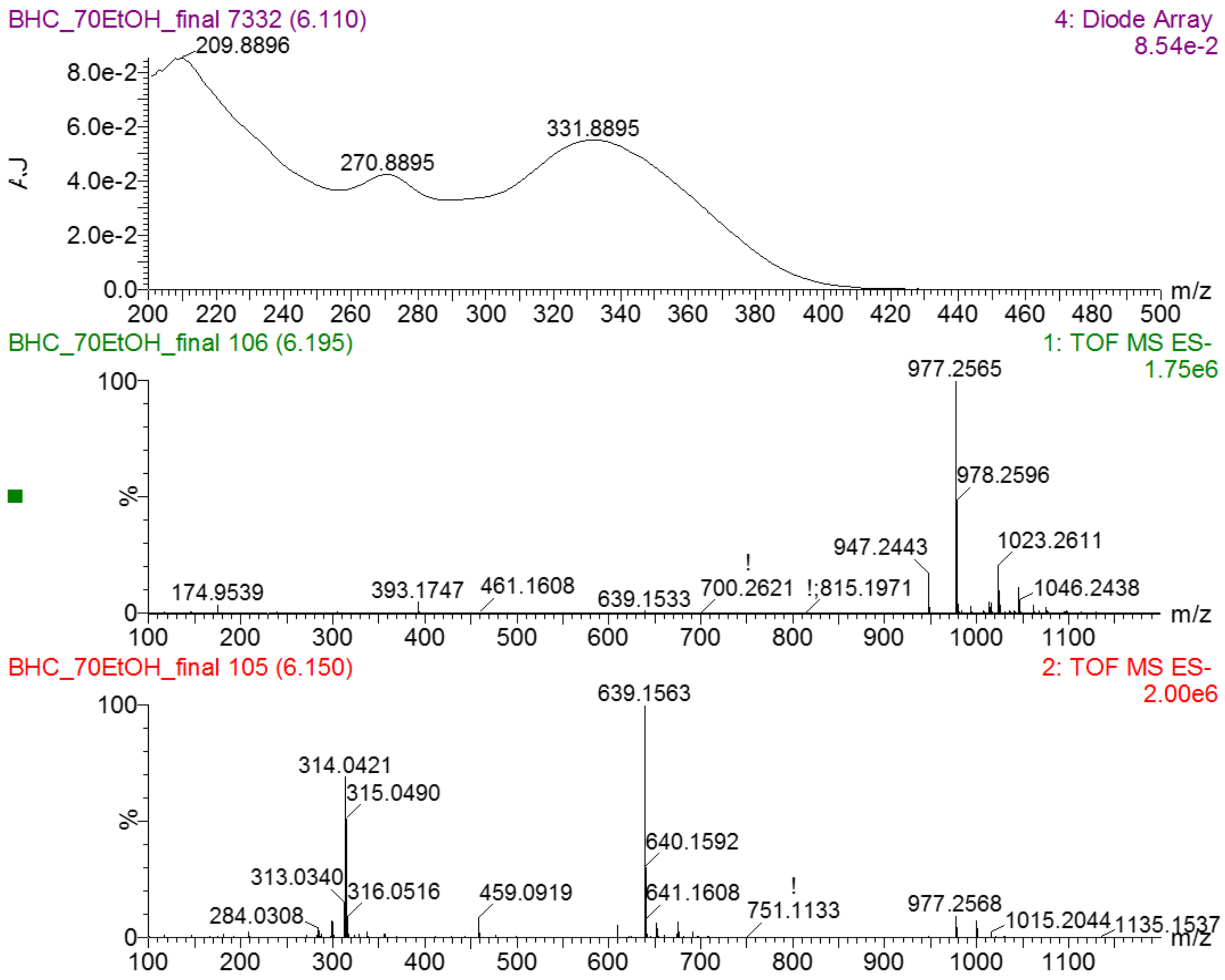

[Elemental Composition]

Single Mass Analysis

Tolerance = 10.0 PPM / DBE: min = -1.5, max = 50.0

Element prediction: Off

Number of isotope peaks used for i-FIT = 3

Monoisotopic Mass, Even Electron Ions

420 formula(e) evaluated with 7 results within limits (up to 50 best isotopic matches for each mass)

Elements Used:

Mass	Calc. Mass	mDa	PPM	DBE	Formula	i-FIT	i-FIT Norm	Fit Conf %	C	H	O
977.2565	977.2563	0.2	0.2	20.5	C44 H49 O25	660.4	0.001	99.94	44	49	25
	977.2622	-5.7	-5.8	11.5	C37 H53 O30	668.2	7.761	0.04	37	53	30
	977.2504	6.1	6.2	29.5	C51 H45 O20	669.2	8.784	0.02	51	45	20
	977.2528	3.7	3.8	-1.5	C26 H57 O38	671.0	10.593	0.00	26	57	38
	977.2469	9.6	9.8	7.5	C33 H53 O33	671.4	10.956	0.00	33	53	33
	977.2598	-3.3	-3.4	42.5	C62 H41 O12	672.3	11.865	0.00	62	41	12
	977.2657	-9.2	-9.4	33.5	C55 H45 O17	672.3	11.929	0.00	55	45	17
